# The effect of mild hypothermia plus rutin on the treatment of spinal cord injury and inflammatory factors by repressing TGF-β/smad pathway

**DOI:** 10.1590/ACB360307

**Published:** 2021-05-07

**Authors:** Shudan Yao, Lihang Wang, Qiling Chen, Tingsheng Lu, Xingwei Pu, Chunshan Luo

**Affiliations:** 1 MM. Department of Spine Surgery – Guizhou Orthopedic Hospital – Guizhou Province, China.; 2 PhD. Department of Spine Surgery – Guizhou Orthopedic Hospital – Guizhou Province, China.

**Keywords:** Rutin, Induced Hypothermia, Spinal Cord Injuries, Inflammation, Rats

## Abstract

**Purpose:**

To probe the mechanism of mild hypothermia combined with rutin in the treatment of spinal cord injury (SCI).

**Methods:**

Thirty rats were randomized into the following groups: control, sham, model, mild hypothermia (MH), and mild hypothermia plus rutin (MH+Rutin). We used modified Allen’s method to injure the spinal cord (T10) in rats, and then treated it with MH or/and rutin immediately. BBB scores were performed on all rats. We used HE staining for observing the injured spinal cord tissue; ELISA for assaying TNF-α, IL-1β, IL-8, Myeloperoxidase (MPO), and Malondialdehyde (MDA) contents; Dihydroethidium (DHE) for measuring the reactive oxygen species (ROS) content; flow cytometry for detecting apoptosis; and both RT-qPCR and Western blot for determining the expression levels of TGF-β/Smad pathway related proteins (TGF-β, Smad2, and Smad3).

**Results:**

In comparison with model group, the BBB score of MH increased to a certain extent and MH+Rutin group increased more than MH group (p < 0.05). After treatment with MH and MH+Rutin, the inflammatory infiltration diminished. MH and MH+Rutin tellingly dwindled TNF-β, MDA and ROS contents (p < 0.01), and minified spinal cord cell apoptosis. MH and MH+Rutin could patently diminished TGF-β1, Smad2, and Smad3 expression (p < 0.01).

**Conclusions:**

MH+Rutin can suppress the activation of TGF-β/Smad pathway, hence repressing the cellular inflammatory response after SCI.

## Introduction

Spinal cord injury (SCI), a devastating illness, is principally induced by falls and road traffic accidents, and can have a profound impact on the patient’s health and emotions, as well as a huge economic burden for the family and the entire society^1^. In addition to the direct consequence of the loss of motor, sensory, and autonomic nervous system function, the secondary protrusions at the injured site can aggravate the injury. Subsequent problems include muscular atrophy, chronic pain, urinary tract infections, and pressure sores. Statistical data manifested that 930.000 SCI patients were newly added in 2016. Thus, SCI is mountingly regarded as a focus impacting global health^2^. The damage associate with SCI is classified into two stages: immediate and irreversible main damage, and the secondary damage, caused by diverse chemical substances, embracing free radicals and excitatory amino acids, which are the pivotal factors of motor and sensory defects^3^.

Systemic hypothermia has been broadly applied in the field of SCI research. In clinical trials, particularly in remedying neonatal asphyxia, cardiac arrest, and acute SCI, surface cooling can be carried out by utilizing ice blankets, cooling bags or soaking in an ice bath, so as to realize systemic cryotherapy^4^. Clinical evidence has illuminated that the hypothermia in patients with severe acute SCI does not dramatically escalate the risk of complications^5^,^6^. The study outcomes displayed that athletes have attained a good prognosis by applying hypothermia treatment early after severe cervical SCI^7^. Research has expounded that hypothermia can reduce secondary pathological mechanisms, such as apoptosis, edema, oxidative stress, excitotoxicity, inflammation, etc.^4^.

Flavonoid is the main dietary component of plant-based food and is bioactive polyphenols with anti-inflammatory and antioxidant effects. Bone health is related to the consumption of flavonoids, whose intake increases bone density (BMD) in the neck and spine, as well as decreases bone resorption in perimenopausal women^8^. Supposedly, flavonoids reduce low-level inflammation and oxidative stress as markers of protection against bone loss. In addition, flavonoids are believed to promote the upregulation of osteoblast activity signaling pathways^9^. Rutin is a flavonolid-type polyphenol, which is composed of the flavonol quercetin and the disaccharide rutinose. Rutin has anti-tumor, antibacterial and other biological effects, mainly related to antioxidant and anti-inflammatory activities. Rutin was showed to promote ossification in bone cells as well as to possess punctuate proliferative activity with minimal influence to cell cycle distribution.

Hereby, this investigation was aimed at delving into the treatment effect and related mechanisms of mild hypothermia plus rutin on SCI, so as to offering theoretical basis and potential therapy for remedying SCI.

## Methods

The animal experiment research protocol was approved by the Ethics Committee of Guizhou Orthopedic Hospital and performed in accordance with the *Guidelines for the Care and Use of Experimental Animals*. The experiment was also approved by the Ethics Committee of the Guizhou Orthopedic Hospital (NO.t-a309).

### Construction and processing of spinal cordinjury model

The rat spinal cord injury model provides an important mammalian model for evaluating treatment strategies and understanding the pathological basis of spinal cord injury. We anesthetized the rats via intraperitoneal injection of 10% chloral hydrate, shaved their back hair, and disinfected the skin with iodophor, subsequently cutting the skin longitudinally with the T11 thoracic spine as the midpoint, about 3 to 4 cm. After incising the skin, we removed the muscles on the T10 and T11 thoracic vertebrae, and exposed the T10 and T11 lamina. Then, we used small scissors and hemostatic forceps to carefully remove the T10 and T11 lamina, exposed the spinal cord, and gently sticked the self-made pad on the exposed epidural surface. Subsequently, we used a 12 g percussion stick, a self-made percussion device, to hit the gasket vertically, from a 7 mm height free fall from the graduated sleeve^10^-^11^. By using this method, ruptured middle spinal cord, intramedullary hemorrhage, and reperfusion were observed, which caused acute SCI. Complete spinal cord injury causes permanent paralysis of the hindlimbs and a corresponding impairment of the sensory and autonomic system functions. The gasket was stressed and damaged the spinal cord. Ultimately, the muscles, fascia, and skin were sutured, and the wound was disinfected again.

### Grouping

Thirty healthy adult female SD rats, weighing 220–250 g (Hunan SJA Laboratory Animal Co.Ltd), were divided into the following groups: control, sham, model, mild hypothermia (MH), and mild hypothermia + rutin (MH+Rutin), with 6 rats in each group. Subsequently, we supplemented some BBB scores of the mice in the MH (total n = 15) and the MH+Rutin groups (total n = 15). Among them, control group was composed by normal reared rats; sham group, by rats with simple operation, but not hitting spinal cord; and model group, by SCI model rats. In MH group, after modelling, we placed the rats on an ice blanket to cool down. We maintained their rectal temperature at 28–32 ºC lasting 4 h, and then rewarmed them. In another hand, in MH+Rutin group, after modelling, we also placed the rats on an ice blanket to cool down, treating them, afterwards, with intraperitoneal injection of rutin (30 mg/kg, once daily, for 3 days; Solarbio), and controlling their rectal temperature at 28–32 ºC lasting 4 h; then, we rewarmed the rats. BBB scores were performed on all rats at 24 h, 48 h, 72 h, 7 days, and 30 days subsequent to surgery. Rats in each group were sacrificed 72 h or 7 days later, and spinal cord tissues were taken for subsequent studies.

### HE staining

The tissues were taken and irrigated with running water for several hours. Then, they were dehydrated by 70, 80, and 90% ethanol solutions, and placed into a mixture of pure alcohol and xylene (1:1), lasting 15 min. Subsequently, the tissues were dewaxed utilizing xylene I, lasting 15 min, and xylene II, lasting the same amount of time (until transparent). We added the mixture of xylene and paraffin (1:1) for 15 min, then paraffins I and II for 50–60 min each. Next, paraffin embedding and slicing were conducted. The paraffin slices were baked, then dewaxed and hydrated. We placed the slices into the distilled water, and then into hematoxylin aqueous solution (ZLI-9610; ZSGB-Bio) for staining, lasting 3 min. Later, the slices were differentiated into a hydrochloric acid ethanol solution, lasting 15 s. Plus, they were irrigated with water; backed to blue, using Scott Blue Solution (G1865; Solarbio), for 15 s; rinsed with running water; stained with eosin lasting solution (G1100; Solarbio), for 3 min; and rinsed with running water. Then, dehydration, transparency, and sealing, microscopic examination were implemented.

### Detection of inflammatory factors

We isolated the rat serum from each group. Later, samples and enzymes were added, and incubation, mixing, washing, color development and termination were conducted, in sequence, according to the ELISA kit’s instructions for detecting TNF-a (m1002859, enzyme-linked), IL-1β (m1003549, enzyme-linked), IL-8 (m1037351, enzyme-linked), and Myeloperoxidase (MPO) (m1003250, enzyme-linked). The OD value was tested at 450 nm, by using multifunctional microplate reader (S/N502000011, TECAN).We calculated the linear regression equation of the standard curve with the concentration of the standard substance and the OD value, substituting the OD value of the sample into the equation, to calculate the sample concentration, and multiplying it by the dilution factor, to obtain the actual concentration of the sample.

### Detection of ROS and MDA content in spinalcord tissue

Seventy-two hours after SCI, reactive oxygen species (ROS) production in spinal cord samples was measured by the oxidative fluorescent dye dihydroethidine (DHE). About 10 µm of spinal cord frozen section were washed with PBS at 37 °C for half an hour, then incubated with DHE for another half hour at 37 °C. The oxidized DHE was measured via fluorescence microscopy. Seventy-two hours after SCI, 1 cm of spinal cord tissue was taken with the injured segment as the center. After tissue homogenization, we detected Malondialdehyde (MDA) content (nmol/mg), as per kit instructions.

### Flow cytometry

The spinal cord of the rats in each group was cut and digested into single-cell suspensions by trypsin. After washing with PBS for 3 times, the cell density was adjusted to 5×10^5^ cells/mL. The cells were incubated with Annexin V and PI, lasting 30 min at 4 °C, in the dark. Subsequent to the incubation, we washed the cells with PBS for 3 times, adjusted the cell density to 10^6^ cells/mL for detection with flow cytometry (C6), and used CFlow Plus software for data analysis.

### RT-qPCR

The spinal cord tissue was ground into powder with liquid nitrogen, and an appropriate amount of TRlzon lysis solution (CW0580S; CWBIO) was added. After that, the powder was converted into a liquid state to extract total RNA. We measured the concentration and purity of RNA (OD260/OD280) with UV-Vis spectrophotometer. The RNA was synthesized by reverse transcription, by applying the HiFiScript cDNA first-strand synthesis kit (CW2569M; CWBIO). We used fluorescent PCR instrument (CFX Connect™ real-time; Bio-Rad Laboratories) for performing RT-qPCR. The reaction system was: RNase Free dH2O 9.5 µL; cDNA 1 µL; upstream primer 1 µL; downstream primer 1 µL; and 2×qPCR mixture 12.5 µL. The reaction steps were: pre-denaturation 95 °C, 10 min; denaturation 95 °C,10 s; annealing 58 °C, 30 s; extension 72 °C, 30 s; 40 cycles. The primer sequences are listed in Table 1 (synthesized by General Biosystems [Anhui] Co., Ltd.), using β-actin as internal reference. The relative expression of TGF-β1, Smad3, and Smad2 were calculated as per 2^-△△Ct^ method.

### Western blot

We cut 50 mg of sample and put it into a centrifuge tube. Then, it was added 1 mL of RIPA cell lysate (C1053; APPLYGEN). Afterwards, we ground it thoroughly in a grinder (65HZ, 60s) to a homogenate; centrifuged it at 12.000 rpm, lasting 15 min; and carefully aspirated the supernatant, to obtain the total protein. We assayed the protein concentration as per BCA Protein Assay Kit (CW0014S; CWBIO). Subsequently, the protein was denatured, loaded, and subjected to SDS-PAGE, lasting 2 h. Then, it was transferred to the PVDF Membrane (IPVH00010; Millipore), with a constant current of 300 mA, lasting 80 min. We incubated the membrane with primary antibodies—anti-TGF-β1 (bs-0068R, Bioss, 1/500), anti-Smad2 (AF6449, Affinity, 1/1000), anti-Smad3 (ab40854, Abcam, 1/1000), and anti-GAPDH (TA-08, ZSGB-Bio, 1/2000)—overnight at 4 °C; then with HRP-labeled secondary antibodies—HRP-labeled goat anti-mouse IgG (H+L) (ZB-2305, ZSGB-Bio, 1/2000), HRP-labeled goat anti-rabbit IgG (H+L) (ZB-2301, ZSGB-Bio, 1/2000)—, lasting 2 h, at indoor temperature. ECL luminescent liquid (RJ239676; Thermo Fisher Scientific) was dropped onto the membrane and exposed in a gel imaging system. Ultimately, we used Quantityone software for analyzing the gray value of each antibody band.

### Statistical analysis

All data were statistically analyzed with Graphpad Prism7 and expressed as mean ± SD. The inter-group significant differences were analyzed via T-test, one-way and two-way ANOVA, and *p < 0.05.

## Results

### Motor function scores

The motor function scores of the five groups at different times after surgery are provided in Table 1. BBB scores of the model group at different time points were strikingly lower than those of the control group (p < 0.05). Besides, in comparison to the model group, the BBB scores of the MH group increased to a certain extent, and the ones of the MH+Rutin group increased more than those of the MH group (p < 0.05). The BBB scores of MH+Rutin group increased significantly at 30 days (p < 0.05).

**Table 1:** Primer sequences of TGF-β1, Smad3, Smad2, and β-actin.

Primer	Sequence
TGF-β1 F	GCTGAACCAAGGAGACGGAA
TGF-β1 R	GAAGTTGGCATGGTAGCCCT
Smad3 F	GCACACAATAACTTGGACCTACA
Smad3 R	CAGTTGGGAGACTGGACGAA
Smad2 F	TGAACTGTCTCCTACCACTCTCT
Smad2 R	CACTCCCCTTCCTATATGCCTTCTG
β-actin F	GCCATGTACGTAGCCATCCA
β-actin R	GAACCGCTCATTGCCGATAG

### Pathological alterations of spinal cord tissue

**Figure 1:**
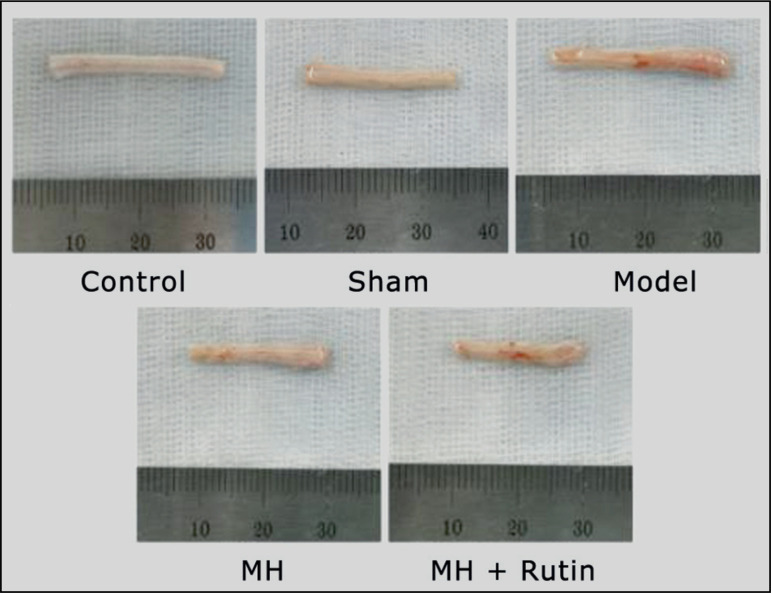
Spinal cord of each group 7 days after surgery. MH: mild hypothermia; MH+Rutin: mild hypothermia plus rutin.

**Figure 2:**
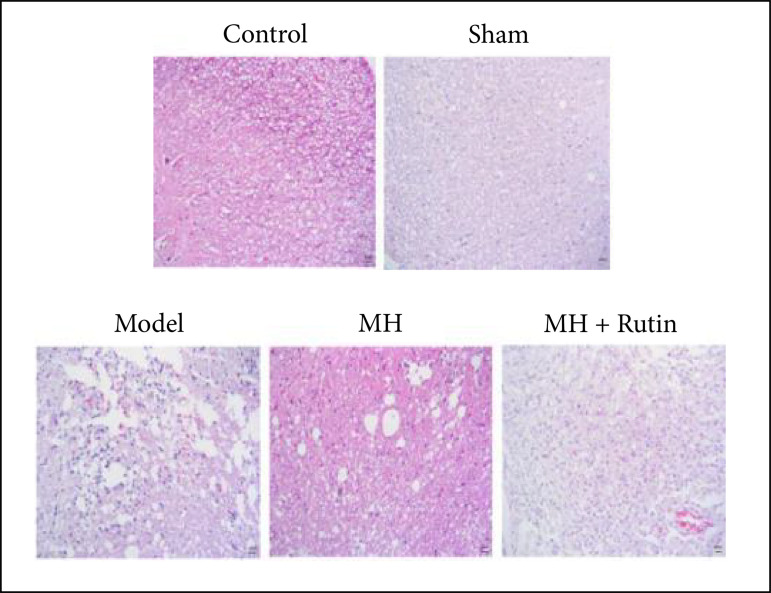
HE staining to observe the pathological changes of spinal cord. MH: mild hypothermia; MH+Rutin: mild hypothermia plus rutin.

The spinal cord of the five groups, 7 days after surgery, is shown in Fig. 1. The spinal cords of the control and sham groups were more uniform, and their surfaces were smooth; whereas, in the model, MH, and MH+Rutin groups, the morphology of the spinal cords has changed to a certain extent, and the size of the front and back sides was different. It can be seen, under high power microscope by HE staining, that the structure of the spinal cord tissue in the control and sham groups was clear, without obvious cavities and hemorrhage. Also, no inflammatory cell infiltration was observed in the tissue, and the nerve cells were normal in shape and neatly arranged. In the spinal cord injury (SCI) model group, massive hemorrhage, cell swelling, cell vacuolar degeneration, partial nucleus shrinkage, disordered arrangement, and a large number of inflammatory cell infiltration occurred. After MH and MH+Rutin treatment, cell vacuolar degeneration was reduced, the morphology of nerve cells was effectively restored, the arrangement was relatively complete, and the inflammation infiltration was reduced (Fig. 2).

### Influence of MH+Rutin on inflammatory response in SCI

**Figure 3:**
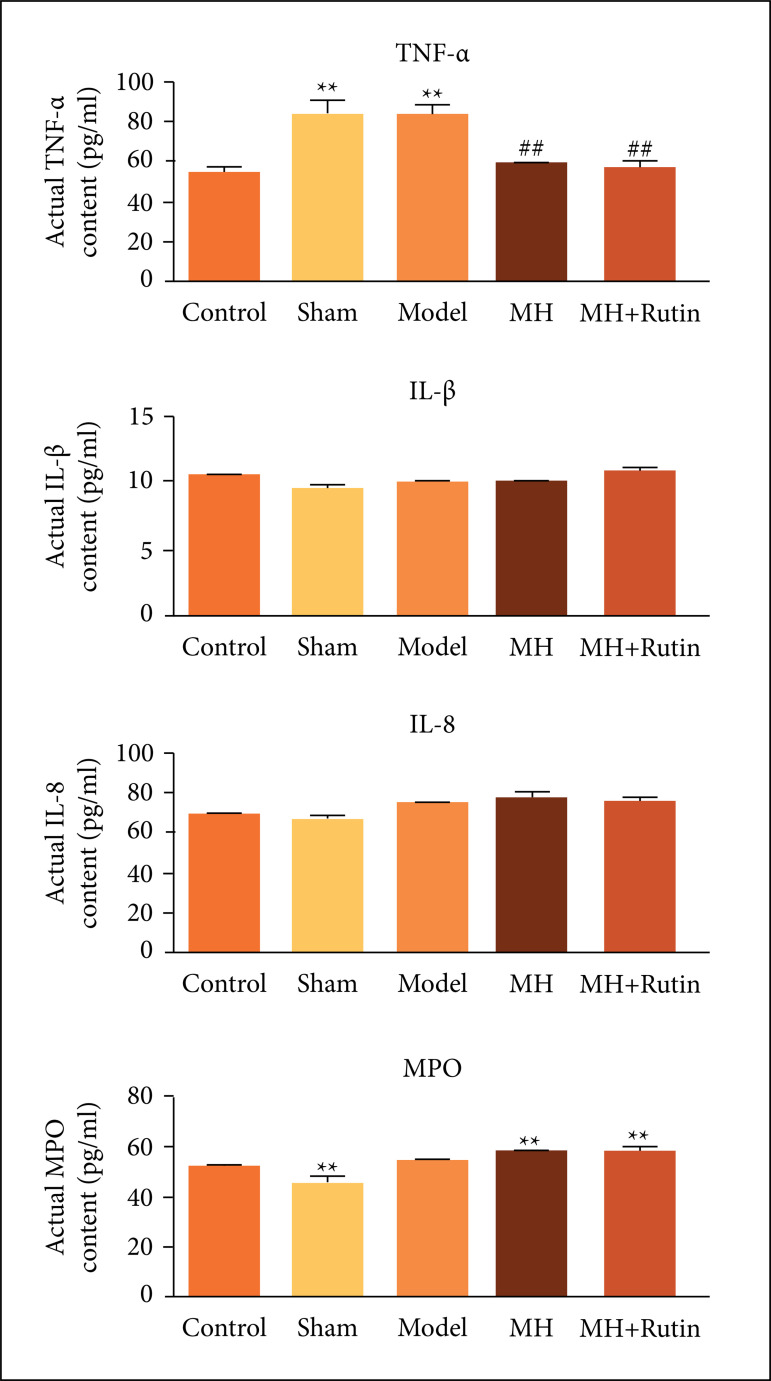
ELISA to detect TNF-a, IL-1β, IL-8, and MPO contents. **p < 0.01 *vs.* control group; ##p < 0.01 vs. model group; MH: mild hypothermia; MH+Rutin: mild hypothermia plus rutin.

For inquiring into the changes of inflammatory response during the treatment of SCI with MH+Rutin, our personnel used ELISA for examining inflammatory factor (TNF-a, IL-1β, and IL-8) and MPO contents. As a result, TNF-a content was sensibly higher in the sham and model groups than in the control group (p < 0.01), whereas MH and MH+Rutin plainly minified TNF-a content (p < 0.01). Furthermore, IL-1β and IL-8 contents did not alter dramatically in the five groups. Additionally, MPO content was patently lower in the sham group than in the control one (p < 0.01). Compared with the model group, MH and MH+Rutin augmented MPO content (p > 0.05) (Fig. 3).

### Influence of MH+Rutin on oxidative stress in SCI

**Figure 4:**
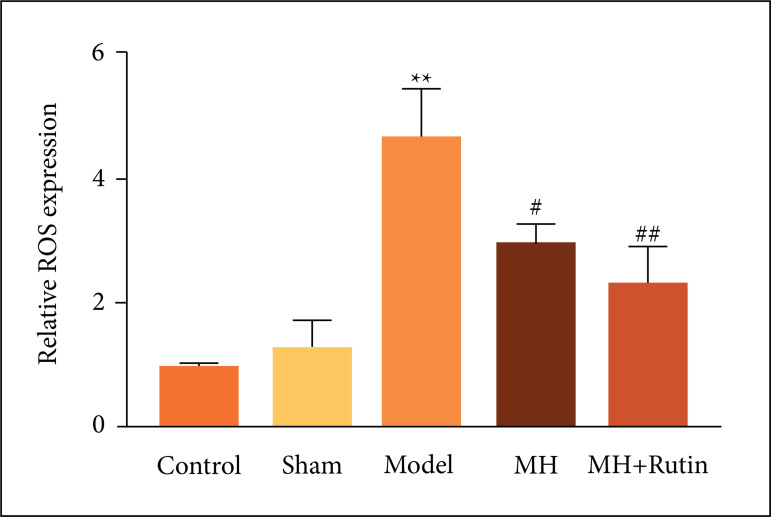
Relative ROS expression in rat spinal cord tissue.**p < 0.01 *vs.* control group; #p < 0.05 and ##p < 0.01 vs. model group; MH: mild hypothermia; MH+Rutin: mild hypothermia plus rutin.

**Figure 5:**
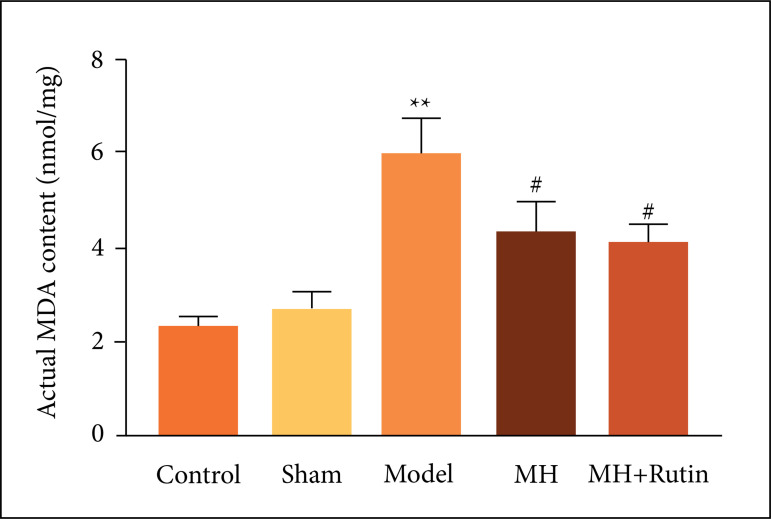
ELISA to detect TNF-a, IL-1β, IL-8, and MPO contents. **p < 0.01 *vs.* control group; #p < 0.05 vs. model group; MH: mild hypothermia; MH+Rutin: mild hypothermia plus rutin.

For exploring the changes of oxidative stress during the treatment of SCI with MH+Rutin, we examined ROS and MDA contents in spinal cord tissue, respectively. ROS content in the model group was significantly higher than in the sham and control groups (p < 0.01), whereas MH and MH+Rutin dramatically dwindled ROS content (p < 0.01) (Fig. 4). MDA content was perspicuously higher in the model group than in the sham and control groups (p < 0.01), whilst MH and MH+Rutin conspicuously diminished MDA content (p < 0.05) (Fig. 5).

### Influence of MH+Rutin on apoptosis after SCI

**Figure 6:**
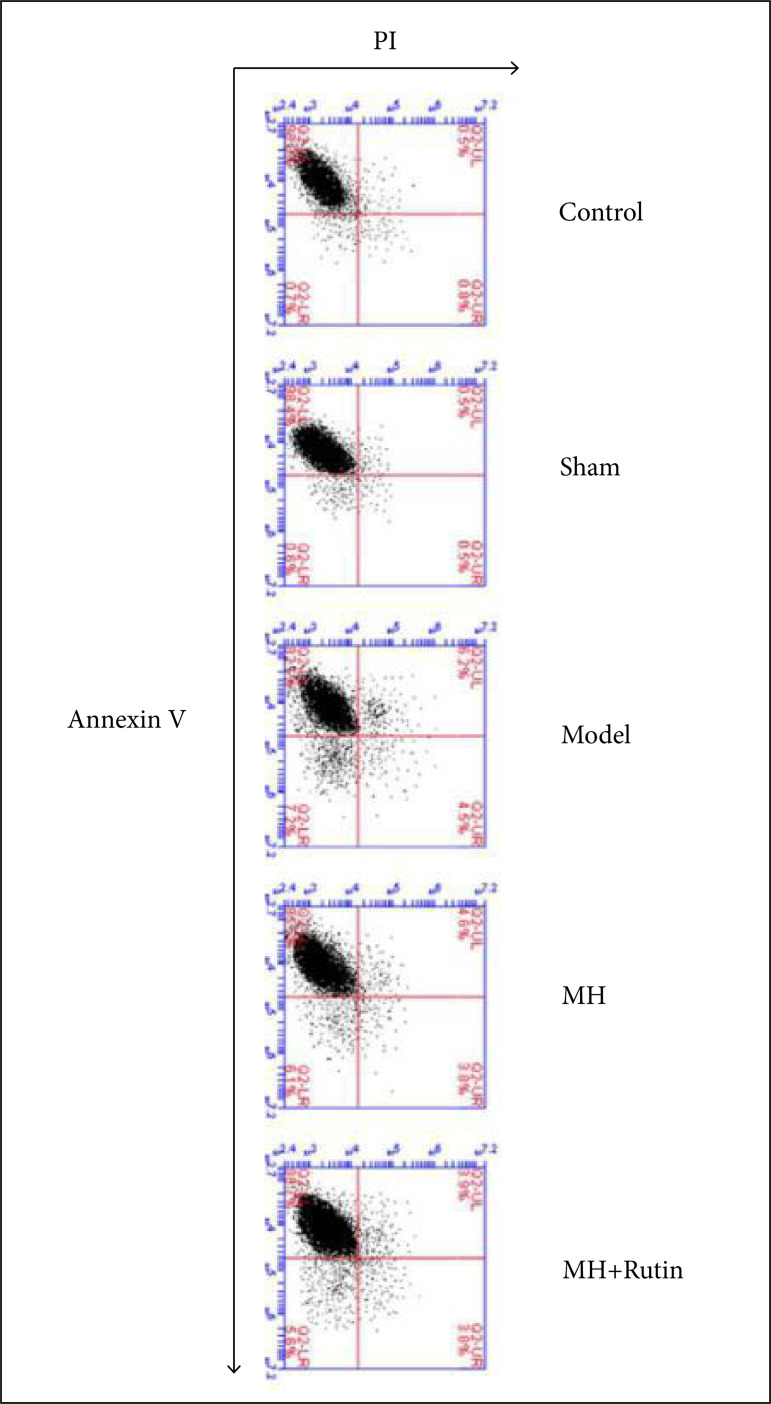
Flow cytometry to detect apoptosis. MH: mild hypothermia; MH+Rutin: mild hypothermia plus rutin.

To probe whether MH+Rutin would or not affect cell apoptosis after SCI, we used flow cytometry for testing spinal cord cell apoptosis. Consequently, the apoptosis rates in the control and sham groups were 0.7% ± 0.3%, and 0.6% ± 0.4%, respectively. In another hand, the rate in the model group was 7.1% ± 1.3%; and, in MH and MH+Rutin groups, 6.1% ± 0.8% and 5.6% ± 0.7%, respectively, which were rates perspicuously lower than those of the model group (p < 0.05) (Fig. 6). The aforementioned results revealed that MH+Rutin can repress cell apoptosis after SCI.

### Influence of MH+Rutin on TGF-β/Smad pathway in SCI

**Figure 7:**
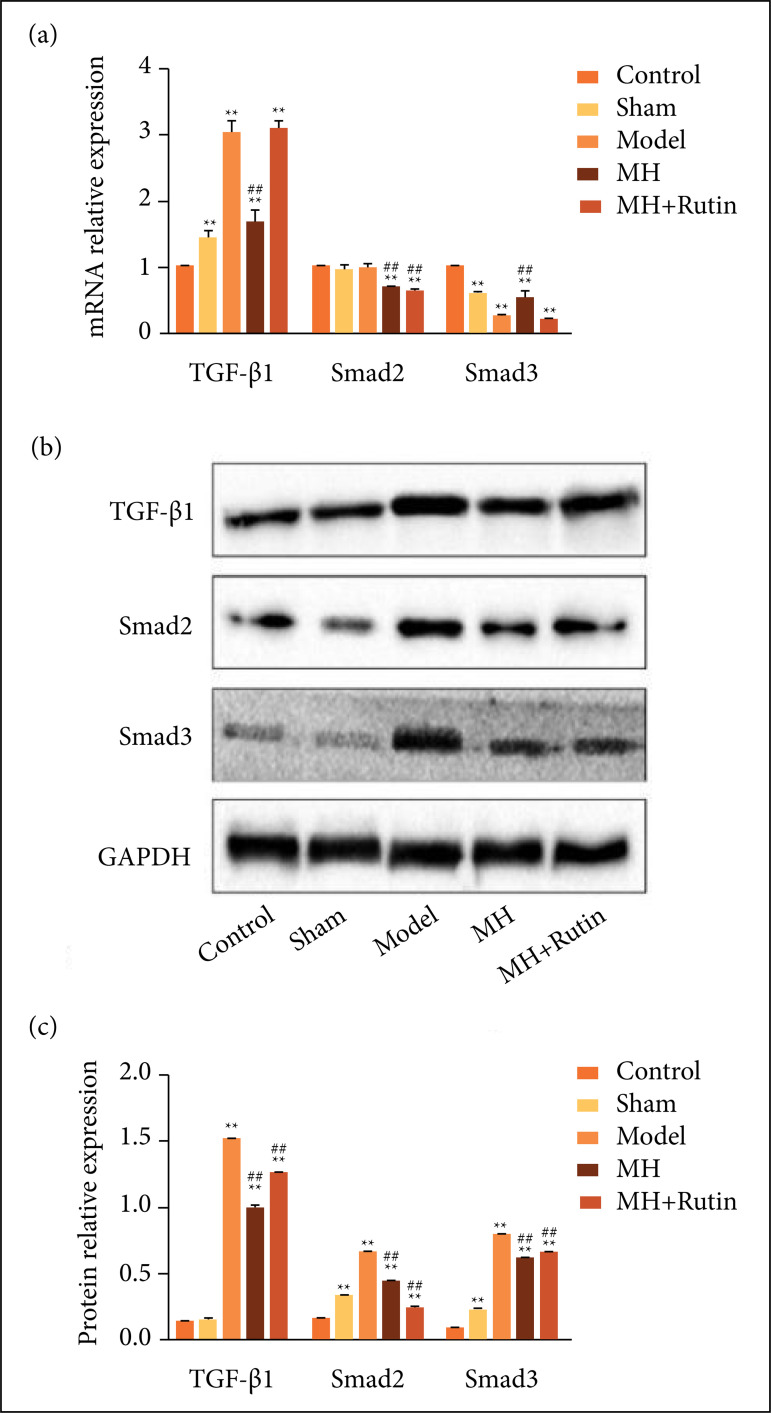
RT-qPCR and Western blot to detect the expression of TGF-beta/Smad pathway related molecules. **(a)** RT-qPCR to assay TGF-β1, Smad2, and Smad3 gene expression; **(b–c)** Western blot to assay TGF-β1, Smad2, and Smad3 protein expression. **p < 0.01 *vs.* control group; ##p < 0.01 vs. model group; MH: mild hypothermia; MH+Rutin: mild hypothermia plus rutin.

To inquire whether the TGF-β/Smad pathway was involved or not in the treatment of SCI with MH+Rutin, we adopted RT-qPCR and Western blot for assaying the mRNA and protein expression levels of pathway related molecules (TGF-β1, Smad2, and Smad3). RT-qPCR revealed that in comparison with the control group, TGF-β1 mRNA level in the model group was saliently augmented (p < 0.01), whereas MH noticeably diminished TGF-β1 expression (p < 0.01). In contrast to the model group, MH and MH+Rutin strikingly diminished Smad2 mRNA level (p < 0.01). Besides, Smad3 mRNA level was significantly lower in model group than in the control one(p < 0.01). MH significantly augmented Smad3 expression(p < 0.01) (Fig. 7a). Western blot uncovered that, in comparison to the control group, TGF-β1, Smad2, and Smad3 protein levels in the model group were plainly increased(p < 0.01), and MH and MH+Rutin could significantly reduce TGF-β1, Smad2, and Smad3 protein levels(p < 0.01) (Figs. 7b and c). Those results evinced that MH+Rutin can suppress the activation of TGF-β/Smad pathway, thereby restraining the cellular inflammatory response after SCI.

### Discussion

In recent years, mild hypothermia has been considered a weighty method of neuroprotection. It can improve the function of the cerebral cortex; inhibit cerebral oxygen metabolism^12^; protect the blood-brain barrier^13^; inhibit the excessive release of neurotransmitters^12^, inflammatory response, and calcium overload^14^; as well as accelerate the growth of nerve cell trunk; and inhibit neuronal apoptosis^15^, thereby alleviating and improving nervous system damage^16^. Zhang *et al*.^4^ have uncovered that hypothermia treatment can effectively reduce the increase of extracellular ascorbic acid concentration caused by acute SCI, thus reducing secondary SCI, and promoting spinal cord repair. In this study, after treating SCI with mild hypothermia, the structure and morphology of the rat spinal cord tissues were effectively protected, the arrangement was relatively complete, and the inflammatory infiltration was reduced. In addition, TNF-a content was significantly reduced; MPO content was partially increased; and ROS and MDA content decreased. The aforementioned implied that mild hypothermia may mitigate nervous system damage by reducing inflammation and oxidative stress.

Rutin, also known as rutinoside, is a glycoside composed of the flavonoid aglycone quercetin and the disaccharide rutinose, and is a citrus flavonoid glycoside found in buckwheat. Rutin has been substantiated to have diverse pharmacological activities, and serves as an antioxidant, as well as cytoprotective, vasoprotective, anticancer, neuroprotective, and cardioprotective agents^18^-^20^. Khan *et al*.^21^ have discovered that ischemia-reperfusion injury, caused the production of free radicals, hence posing nerve damage and taking rutin, can reduce p53 expression to prevent morphological changes and increase the enzyme activity of endogenous antioxidants to reduce ischemic nervous cell apoptosis. Wu *et al*.^22^ have corroborated that rutin can significantly reduce reactive oxygen species, malondialdehyde, NLRP3, ASC, caspase-1, IL-1β, IL-18, and TNF-a levels in rat SCI models, as well as attenuate histological changes and improve exercise recovery. In the present study, MH+Rutin could increase the BBB score of rats; meliorate the structure of spinal cord tissue; significantly reduce TNF-a content; increase MPO content; and reduce ROS and MDA contents in the spinal cord, indicating that the mechanism of rutin may be implicated in alleviating inflammation and oxidative stress.

The activation of microglia and the production of various cytokines after SCI lead to increased inflammatory response and enhanced activation of the TGF-β and Smad2 pathways^23^. The TGF-β/Smad pathway contributes to scar formation, and plays a role in recovering after an injury, but excessive formation of scar tissue affects functional recovery^24^. As a downstream signal of TGF-β, Smad2 seems to mediate scar gliosis by activating the intrinsic transcriptional program of nerve cells. Hellal *et al*.^25^ used paclitaxel to inhibit kinesin-dependent Smad2 translocation and reduce the scar tissue around the lesion. In this study, treating rat SCI with mild hypothermia significantly inhibited TGF-β1 and Smad2 gene expressions, while MH+Rutin had no significant effect on the expression of TGF-β1. This indicates that rutin may inhibit the effect of mild hypothermia on TGF-β1 gene expression, but has no effect on the expression of Smad2. On the contrary, mild hypothermia up-regulated Smad3 gene expression, which rutin could inhibit. At the protein level, the effects of MH+Rutin on TGF-β1 and Smad3 expressions were not significantly different from mild hypothermia, but they exhibited a coordinated effect on Smad2 expression. It is possible that rutin mainly acts by inhibiting this expression on Smad2, indicating that MH+Rutin may inhibit the activation of TGF-β1 and Smad2 in the TGF-β/Smad pathway to inhibit secondary injury after SCI.

## Conclusion

Overall, MH+Rutin is available to inhibit the activation of TGF-β/Smad pathway, thereby inhibiting the cellular inflammatory response after SCI.
